# Technological Advances and Medical Applications of Implantable Electronic Devices: From the Heart, Brain, and Skin to Gastrointestinal Organs

**DOI:** 10.3390/bios15080543

**Published:** 2025-08-18

**Authors:** Jonghyun Lee, Sung Yong Han, Young Woo Kwon

**Affiliations:** 1 Division of Gastroenterology, Biomedical Research Institute, Pusan National University Hospital, Busan 49241, Republic of Korea; 2 Department of Internal Medicine, Pusan National University College of Medicine, Yangsan 44955, Republic of Korea; 3 Inter-University Semiconductor Research Center, Pusan National University, Busan 46241, Republic of Korea

**Keywords:** implantable electronic devices, cardiac implants, brain–machine interface, continuous glucose monitoring, biliary stents, wireless sensors

## Abstract

Implantable electronic devices are driving innovation in modern medical technology and have significantly improved patients’ quality of life. This review comprehensively analyzes the latest technological trends in implantable electronic devices used in major organs, including the heart, brain, and skin. Additionally, it explores the potential for application in the gastrointestinal system, particularly in the field of biliary stents, in which development has been limited. In the cardiac field, wireless pacemakers, subcutaneous implantable cardioverter-defibrillators, and cardiac resynchronization therapy devices have been commercialized, significantly improving survival rates and quality of life of patients with cardiovascular diseases. In the field of brain–neural interfaces, biocompatible flexible electrodes and closed-loop deep brain stimulation have improved treatments of neurological disorders, such as Parkinson’s disease and epilepsy. Skin-implantable devices have revolutionized glucose management in patients with diabetes by integrating continuous glucose monitoring and automated insulin delivery systems. Future development of implantable electronic devices incorporating pressure or pH sensors into biliary stents in the gastrointestinal system may significantly improve the prognosis of patients with bile duct cancer. This review systematically organizes the technological advances and clinical outcomes in each field and provides a comprehensive understanding of implantable electronic devices by suggesting future research directions.

## 1. Introduction

Embedded electronic devices represent a core element of modern electronic technology innovation and refer to data-processing electronic devices designed to perform specific functions [1]. Unlike general-purpose computers, they perform limited functions, such as automotive control or display systems [2], providing cost efficiency and real-time operational capability through customized software and hardware design [3]. These devices integrate mechanical, electrical, and chemical components to achieve performance enhancement, miniaturization, and increased reliability of electronic equipment [4].

Recent advances in nanotechnology and material science have significantly improved the design and fabrication of embedded electronic devices. The development of advanced materials, such as graphene, carbon nanotubes, and polymer nanocomposites [5,6,7], combined with innovative manufacturing methods, such as printed electronics and three-dimensional (3D) printing [8,9], has led to smaller, more efficient, and versatile devices. These technological improvements, along with the development of the Internet of Things and wireless technology, are crucial in maximizing the performance of electronic devices [10].

Implantable electronic devices present new possibilities for electronic device design, particularly for space saving and optimizing power consumption within highly integrated circuits [11,12]. Advances in artificial intelligence technology have addressed energy efficiency and compatibility issues, facilitating integration with embedded electronic devices [13]. Furthermore, convergence research focused on diagnosis and management has been actively conducted during the COVID-19 pandemic [14,15,16].

**Figure 1:**
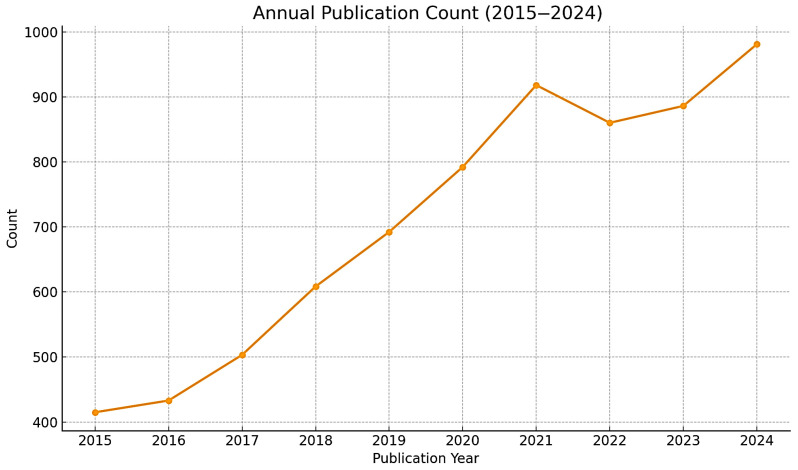
Rising Global Research Output on Implantable Electronic Devices: Annual Publication Trends from 2015 to 2024. Data obtained from Web of Science Core Collection using keywords “implantable electronic device” or “implantable biosensor” or “implantable medical device”. The steady increase from 415 publications in 2015 to 981 in 2024 (CAGR: 10.0%) demonstrates the rapidly growing scientific interest in this field.

The growing academic interest in this field has been demonstrated by analyzing the annual number of publications indexed in the Web of Science database from 2015 to 2024, using related keywords such as ‘implantable electronic device’. As can be seen in Figure 1, the number of publications has increased noticeably over the past decade, from 415 in 2015 to 981 in 2024. This increasing trend highlights the growing interest in research and technological advances worldwide in the development and clinical application of implantable electronic systems.

In the medical field, implantable electronic devices have emerged as an innovative technology [17]. These devices support real-time monitoring of physiological parameters and enable early diagnosis and treatment of diseases. Their clinical applicability has greatly expanded because of advances in wireless power transmission technology and biocompatible sensors [18]. Medical embedded systems are important in patient assessment and health management [19], with devices such as pacemakers, cardiac monitors, and insulin pumps being widely used clinically to improve patients’ quality of life [20,21].

### 1.1. Advantages of Implantable Sensors over Wearable Sensors

Compared to conventional wearable sensors, implantable sensors are attracting attention as a technology that can stably collect signals of high fidelity in internal organs of the human body, which are difficult to access. Sensors located on the surface of the body have limitations in precise measurements due to the low signal-to-noise ratio for pressure, pH, and electrophysiological signals for various reasons [22]. Therefore, it is also difficult to detect micro-signals generated inside the organ in real time. On the other hand, implantable sensors that are directly inserted and fixed into organs can accurately acquire high-resolution bio-signals based on low interfacial impedance and minimized motion artifact characteristics.

The implantable sensor provides high data continuity and reliability by enabling continuous monitoring for a long period of time, regardless of whether the user wears it or not. Furthermore, a closed-loop system integrating sensing, computation, stimulation, or drug delivery functions into a single platform provides a technological foundation for real-time therapeutic intervention beyond diagnosis [23]. Implantable sensors that are stably fixed within the body are not affected by external conditions, resulting in high signal reproducibility and precision. They are advantageous for obtaining “clean” data for long-term observation and prognosis prediction [24]. In addition, they are not exposed outside the skin, offering high aesthetic acceptability and convenience in daily life, and contributing to patient compliance and improved quality of life. Due to these characteristics, implantable sensors are emerging as a core infrastructure for precision medicine beyond simple monitoring, and their expansion into various organ and disease areas is anticipated in the future.

### 1.2. Design Principles for Single Laboratory Fabrication

The development of implantable biomedical electronic devices requires complex technologies. Besides electronic device development, biocompatible packaging is essential for protecting implanted devices from harsh biological environments and extending their lifespans [25]. Since implantable medical devices are inserted into the human body for a long period of time and have the purpose of monitoring bio-signals or performing treatment, a high degree of reliability, safety, and biocompatibility is essential. In a single laboratory environment operated with limited manpower and equipment, it is important to clearly understand and reflect the core components and design principles throughout device manufacturing.

The design of implantable medical devices requires minimizing the overall volume so that they can be fully inserted into tissues, not interfering with physiological functions, and miniaturizing them so that they can be located directly in the area where stimulation and sensing take place [26]. In addition, the ability to selectively stimulate specific tissues, adjust locations, and adapt to changes in stimulation conditions is required. It should be programmable by the therapist and able to send and receive data from external controls or other devices. Intelligent power management technology is essential. Batteries are the main factors limiting miniaturization, and traditional primary batteries are just an alternative option due to problems such as volume, lifetime, difficulty in replacement, and cost [27]. Therefore, it is important to minimize power consumption and maximize efficient power management. In addition, since the device is implanted in the human body, temperature control technology is essential to ensure stable performance without generating excessive heat. Even a temperature increase of just 1–2 °C can cause damage to surrounding tissues or internal components [28].

### 1.3. Overview of Current Implantable Electronic Device Technologies

This review comprehensively analyzes the latest technological trends in implantable electronic devices used in major parts of the human body, including the heart, brain, and skin. It also explores the possibility of expanding these technologies into the gastrointestinal system, particularly in the hepatobiliary field, in which development remains limited. Biliary obstruction can lead to serious complications, and metal stents play a critical role in addressing its management [29,30]. Various sensors and electronic devices can be integrated into stents to perform functions such as monitoring real-time pressure or detecting inflammation.

To provide readers with a comprehensive overview of the current state of implantable electronic device technology, Table 1 presents a detailed comparison of various devices across different organ systems. This table summarizes key specifications including device dimensions, target signals, materials, clinical status, and performance metrics for cardiac, neural, metabolic, and gastrointestinal applications. As shown in Table 1, modern implantable devices have achieved remarkable miniaturization (ranging from micrometers to a few centimeters), extended operational lifespans (from days to over a decade), and reached high-precision sensing capabilities (with accuracy levels suitable for clinical decision-making). These technological achievements form the foundation for the detailed discussions in the following sections.

**Table 1:** Comprehensive Overview of Implantable Electronic Devices: Specifications and Clinical Applications.

Device Category Device Name/Description		Target Organ/Location Size/Dimensions Target Signals Materials	Clinical Status	Performance Metrics	References
Cardiac Devices	Micra Leadless Pacemaker	Right ventricle, 25.9 × 6.7 mm, 2.0 g ECG, R-wave, Titanium, Nitinol	FDA approved (2016)	Battery: 12–17 years, Capture threshold: <1.25 V@0.24 ms, R-wave: 10.7 ± 5.0 mV	[31,32]
	S-ICD (Boston Scientific)	Subcutaneous 83 × 69 × 12.7 mm Surface ECG Titanium	FDA approved (2012)	Shock success: >98%; Battery: 7.5 years; Detection: 170–250 bpm	[33,34]
	CardioMEMS HF System	Pulmonary artery 15 × 3.5 × 2 mm Pressure Nitinol, Fused silica	FDA approved (2014)	Accuracy: ±2 mmHg; Range: 0–50 mmHg; Wireless: 1.5 m	[35,36]
	Implantable Loop Recorder (ICM)	Subcutaneous chest 44 × 7 × 4 mm ECG(long-term) Titanium, Polymer	FDA approved	Battery ~3 years; ECG storage ~59 min; AF detection sensitivity ≈ 95% (clinical performance overview).	[37]
Neural Interfaces	Utah Array	Motor cortex 4 × 4 mm, 96 channels Action potentials, Silicon, Parylene-C	Clinical trials	SNR: >5, Impedance: 30–70 kΩ, Bandwidth: 0.3–7.5 kHz	[38]
	Stentrode (Synchron)	Jugular vein to brain 8 mm diameter, 40 mm length ECoG Nitinol, Platinum	Clinical trials (SWITCH)	Channels: 16, Bandwidth: 0.1–10 kHz, 12-month safety	[39]
	DBS Electrodes (Medtronic)	Subthalamic nucleus 1.27 mm diameter, 4 contacts LFP, Beta oscillations Platinum-Iridium	FDA approved	Frequency: 60–185 Hz, Voltage: 0–10.5 V, Pulse width: 60–450 μs	[40,41]
	Neuralink N1	Cerebral cortex 23 × 8 mm chip, 1024 channels Spike activity Flexible polymer threads	Clinical trial (PRIME)	Threads: 64 per chip, Bandwidth: 20 kHz, Wireless: 10 Mbps	[42]
Metabolic Sensors	Dexcom G7 CGM	Subcutaneous tissue 24 × 11 × 2.5 mm Interstitial glucose Polymer membrane, Enzyme	FDA approved (2022)	MARD: 8.2%, Lag: 3.5 min, Duration: 10 days, Range: 40–400 mg/dL	[43]
	Abbott Libre 3	Subcutaneous arm 21 mm diameter × 2.9 mm Glucose Enzymatic sensor	FDA approved (2022)	MARD: 8.9%, Lag: 1.8 ± 4.8 min, Duration: 14 days	[44]
	Eversense 365	Subcutaneous upper arm 18.3 × 3.5 mm Glucose (fluorescence) Fluoropolymer, Hydrogel	FDA approved (2024)	MARD: 8.8%, Duration: 365 days, Calibration: 1/week	[45]
GI/Biliary Devices	Self-Expandable Metal Stent (Uncovered)	Bile duct 8–10 mm diameter, 40–80 mm length N/A (passive) Nitinol	Clinical use	Patency: 4–6 months, Migration: <5%, Occlusion: 20–30%	[46]
	Self-Expandable Metal Stent (Covered)	Bile duct 8–10 mm diameter, 40–80 mm length N/A (passive) Nitinol + polymer covering	Clinical use	Patency: 6–12 months; Migration: <10%; Occlusion: 10–20%	[47]
	Magnetoelastic Sensor	Biliary stent surface 28 μm thickness Viscosity/Mass Metglas, PDMS, Ferrite	Research	SNR: 10^6^, Detection: 17 cm distance, Sensitivity: 0.1% mass change	[18]
	Wireless pH Sensor	Esophagus/Stomach 26 × 13 mm capsule pH, Temperature Silicon nanowire	Research/ FDA cleared	pH range: 0–14, Accuracy: ±0.1 pH, Battery: 48–96 h	[48]
	Smart Biliary Stent	Bile duct 8–10 mm diameter Pressure, pH, Temperature Nitinol, Flexible sensors	Pre-clinical	Pressure: 0–50 mmHg, pH: 4–9, Wireless: 10 cm range	[49,50]

## 2. Implantable Electronic Devices in the Cardiac Field

### 2.1. Electrophysiological Characteristics of the Heart and the Need for Implantable Devices

Implantable electronic devices are highly developed for the heart. Electronic devices implanted in the heart primarily function as pacemakers or monitoring devices, such as electrocardiograms [51,52]. The heart is a vital organ that acts as a pump in the body by generating and transmitting regular electrical signals [53]. Cardiac cells generate electrical signals through sodium, calcium, and potassium ion channels [54]. This electrical activity is essential for maintaining a normal heart rhythm, and implantable electronic devices can monitor and directly regulate this rhythm. Given that cardiovascular diseases accounted for approximately one-third of global deaths in 2021 [55], continuous monitoring is essential for the management and treatment of heart failure, arrhythmias, and heart attacks. Implantable electronic devices have significantly improved survival rates for patients with cardiovascular disease compared to drug therapy alone [56].

### 2.2. Evolution of Pacemakers

Pacemakers are representative implantable electronic devices that have evolved for over 60 years since their first implantation in 1958 [57,58]. Globally, >1 million units are implanted annually, generating electrical signals to maintain heart rhythms [59]. To treat various arrhythmic diseases, such as bradycardia or atrioventricular block [60], biodegradable materials and leadless models have been developed to address lead-related complications, such as infection or perforation in conventional devices [61,62,63,64].

**Figure 2:**
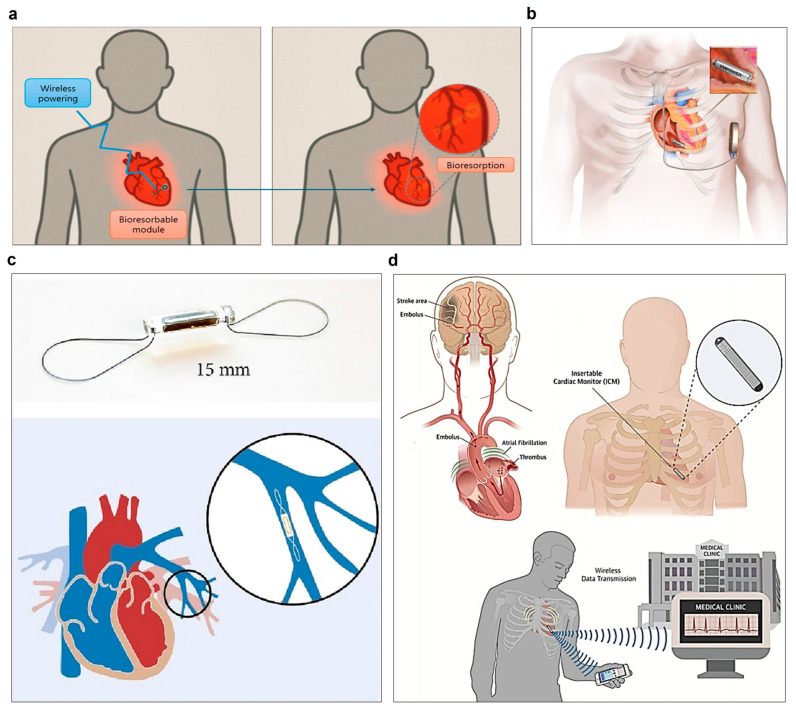
State-of-the-art implantable electronic devices in the cardiac field. (**a**) Operating principle of wireless battery-free pacemaker based on inductive coupling technology operates without battery replacement surgery by receiving power wirelessly from an external source. (**b**) Subcutaneous implantable cardioverter-defibrillator (S-ICD) system demonstrating intercommunicative capabilities with leadless pacing systems; the MODULAR ATP trial investigates this novel approach to prevent vascular complications while enabling anti-tachycardia pacing through wireless communication between devices. Reproduced with permission from [33]. (**c**) Pulmonary artery sensor system for pressure monitoring; the PASSPORT-HF multicenter randomized clinical trial evaluates this technology’s effectiveness in reducing heart failure hospitalizations and improving patient outcomes through continuous hemodynamic monitoring. Reproduced with permission from [35]. (**d**) Smartphone-connected wireless atrial fibrillation diagnostic system presents advanced form of ICM that transmits real-time data to medical staff. Reproduced with permission from [37].

A wireless battery-free pacemaker that uses inductive coupling technology is shown in Figure 2a. This innovative system receives power wirelessly from an external source, eliminating the need for battery replacement surgery and dramatically reducing device size [63].

Zhang et al. developed the world’s smallest wireless biodegradable pacemaker using a galvanic-cell-based bio-power source and near-infrared light guidance [65]. This device is small enough to be inserted into the body using a standard syringe and has successfully demonstrated its heartbeat control function in mouse, pig, dog, and human heart tissue models. Additionally, it is equipped with a closed-loop-based autonomous control system and completely degrades within the body after use, eliminating the need for additional removal surgery. This technology promises various applications in the field of electrical therapy, including neural stimulation, pain management, and post-surgical care for heart surgery.

Limitations and Challenges: Leadless and bioresorbable pacemakers have reduced hardware-related complications, but new clinical and regulatory uncertainties have arisen regarding their long-term management. Battery-free devices powered by inductive coupling must maintain a delicate balance between energy harvesting efficiency and tissue-specific absorption rates, while bioresorbable galvanic batteries may exhibit unpredictable dissolution rates in patients with renal failure or systemic inflammation. Additionally, current non-rechargeable systems lack multi-chamber sensing capabilities, making them difficult to apply in complex conduction disorders, and recovery strategies for non-functional biodegradable devices conflict with post-implantation surveillance obligations, leading to ethical controversies.

### 2.3. Evolution of Implantable Cardioverter-Defibrillators (ICDs)

ICDs are sophisticated devices used for managing life-threatening arrhythmias, such as ventricular tachycardia and ventricular fibrillation [66,67]. These devices treat dangerous ventricular arrhythmias by delivering electrical shocks [68] and providing various therapies, including anti-tachycardia pacing, bradycardia pacing, cardioversion, and defibrillation [69]. Clinical studies have demonstrated that ICDs possess superior efficacy compared with antiarrhythmic drugs in reducing the rate of sudden cardiac death [70].

ICD complications include infection, lead displacement, pulmonary edema, and vascular damage [71]. Therefore, extravascular insertion models, such as subcutaneous ICDs (S-ICDs), have been developed [72,73]. The S-ICD in Figure 2b is positioned only subcutaneously without intravenous leads, fundamentally preventing vascular-related complications. The MODULAR ATP study was the first clinical trial to evaluate the safety and efficacy of a modular cardiac rhythm management system combining a wireless, leadless pacemaker and a subcutaneous implantable cardioverter-defibrillator, verifying the efficacy of anti-tachycardia pacing for ventricular arrhythmias [33]. Among the 151 patients analyzed, the arrhythmia termination rate due to ATP therapy was 61.3%, the device-to-device communication success rate was very high at 98.8%, and no cases of discomfort related to pacing effects were reported [34]. This study suggests that closed-loop electrical therapy via wireless communication between leadless implantable devices is effective in controlling spontaneous arrhythmias and may be utilized as a next-generation arrhythmia treatment strategy.

Limitations and Challenges: Next-generation extravascular ICD platforms mitigate intravascular risks but introduce signal-processing and latency challenges that have not yet been fully solved. Clinical registration data shows that inappropriate shocks have been observed in S-ICD recipients. While modular ATP systems promise wireless anti-tachycardia pacing, bidirectional communication between devices must remain reliable under extreme postural or adiposity-related impedance changes; otherwise, therapy gaps could prove fatal. Lifelong MRI compatibility, battery exchange logistics, and cybersecurity for wireless firmware updates also demand consensus guidelines before widespread adoption.

### 2.4. Other Cardiac Implantable Electronic Devices

Cardiac resynchronization therapy is a treatment that effectively improves cardiac output by correcting ventricular contraction imbalance (asynchrony) caused by conduction disorders.

One of the important developments in heart failure management is the implantable pulmonary artery pressure monitor, which enables real-time assessment of heart status and early treatment [74]. The CardioMEMS sensor shown in Figure 2c wirelessly transmits pulmonary artery pressure data, enabling continuous monitoring of the patient’s hemodynamic status. The PASSPORT-HF multicenter randomized clinical trial is currently evaluating the effectiveness of this sensor system in reducing hospitalization rates and mortality in heart failure patients, and it is expected to contribute improving the timeliness of treatment and prognosis [35].

Additionally, the implantable cardiac monitor (ICM) is useful for early diagnosis of unexplained syncope or intermittent atrial fibrillation through long-term electrocardiogram (ECG) monitoring. The wireless atrial fibrillation diagnostic system shown in Figure 2d enables remote monitoring and personalized cardiac management by transmitting ECG data in real time to a smartphone application [37]. Recently, technology is being developed to integrate wireless pressure sensors into cardiovascular stents, enabling real-time detection of stent closure or stenosis. Such systems offer new possibilities for early detection of complications and improved prognosis following interventional procedures [75,76,77].

### 2.5. Quantitative Performance Metrics

Quantitative characterization results confirmed that modern leadless pacemakers are competitive with traditional systems in terms of both detection accuracy and energy autonomy. In the important Micra AV registration study, the intrinsic R-wave amplitude averaged 10.7 ± 5.0 mV, and the pacemaker capture threshold remained below 1.25 V at 0.24 ms in more than 95% of implant cases, indicating an expected service life of 12 to 17 years under nominal load conditions [31,32]. This data demonstrates that millimeter-scale devices can meet long-term electrophysiological requirements without sacrificing the benefits of miniaturization.

Next-generation defibrillators and wireless hemodynamic monitors enable proactive intervention by providing treatment delays of less than one second and pressure resolution in millimeters. In the MODULAR-ATP study (n = 151), wireless communication between the wireless ventricular pacemaker and S-ICD was successful in 98.8% of ventricular tachycardia suppression pacing commands, and the rate of patients without complications was 97.5% [78].

## 3. Implantable Electronic Devices in Brain–Neural Interface Field

### 3.1. Importance of Brain–Machine Interfaces

In the brain, implantable electronic devices are used as neural stimulators or brainwave monitoring systems that contribute to disease treatment and neuroscientific research [79,80]. These technologies form the foundation of brain–machine interfaces (BMIs), which enable bidirectional communication between neural systems and external devices. Implantable electronic devices in the brain are regarded as core technologies in neural engineering, playing a critical role in decoding and modulating complex neurological signals [38]. They have opened new pathways not only for understanding the fundamental mechanisms of brain function but also for clinical intervention in neurological diseases.

Such devices provide groundbreaking treatment options for a range of disorders, including Parkinson’s disease, epilepsy, chronic motor dysfunction, and spinal cord injury-induced quadriplegia [81,82]. By restoring disrupted neural pathways or bypassing damaged regions through direct electrical stimulation or decoding of cortical activity, they enable motor recovery, seizure suppression, and even volitional control of robotic prostheses. Furthermore, recent developments in closed-loop neuromodulation and AI-assisted signal interpretation are advancing the precision and adaptability of BMIs, offering new hope for personalized neurology.

### 3.2. Hierarchical Structure of Brain Signal Recording Technologies

**Figure 3:**
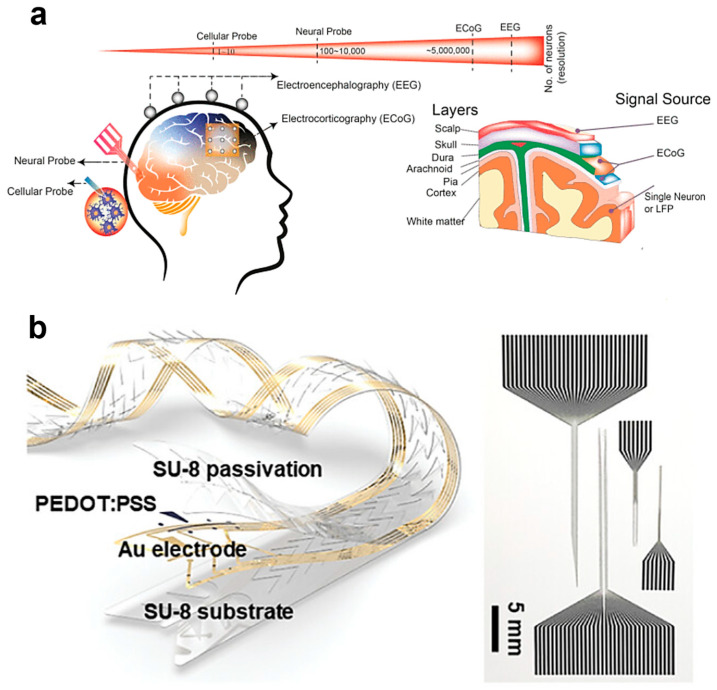
Advances in implantable electronic device technology for brain–neural interfaces. (**a**) Hierarchical organization of brain signal recording technologies. The schematic illustrates neural signal acquisition methods across multiple spatial scales using cellular and neural probes, comparing the invasiveness and resolution of recording modalities from single neuron and LFP recordings to ECoG and EEG. The right panel depicts the layered structure of the brain including scalp, skull, dura, arachnoid, pia, cortex, and white matter, indicating the signal sources for each recording modality. Reproduced with permission from [38]. (**b**) Biomimetic neural probe design for enhanced biocompatibility. The device features a super static interface design composed of PEDOT:PSS-coated Au electrodes on SU-8 substrate, with serpentine architecture providing mechanical flexibility. This design minimizes immune response and enables stable long-term neural interfaces for deep brain signal monitoring and stimulation. Reproduced with permission from [93].

Brain signals are classified into scalp recordings or electroencephalography (EEG), electrocorticography (ECoG), local field potentials (LFPs), and action potentials according to their recording location, each exhibiting different resolutions and levels of invasiveness [83,84,85]. Das et al. comprehensively analyzed and presented the hierarchical structure and practical applications of these technologies (Figure 3a) [38].

EEG is a non-invasive technology employed for seizure management, epilepsy treatment, sleep monitoring, and speech recognition [86,87]; however, it suffers from low transmission rates and signal attenuation issues [88,89]. ECoG, positioned beneath the skull, can record higher-frequency neural signals (1000–2000 Hz) [90,91], making it advantageous for brain–machine interface applications. Implantable neural probes collect LFP signals from deep brain regions, providing detailed information about specific brain areas [38,92].

### 3.3. Next-Generation Distributed and Multichannel Neural Interfaces

Building on these advances in neural signal measurement technology, there has been a paradigm shift toward distributed, multilateral, multichannel, high-channel-count BMI platforms that can investigate and modulate distributed neural circuits in freely behaving animals, beyond single-site probes. These next-generation BMI systems integrate wireless power and data transmission, on-board computation, and miniaturized multi-mode transducers, thereby establishing the foundation for implementing functional links between the cellular level, mesoscale, and behavioral level without physical connections or external cables.

A notable example is the fully implantable, battery-free closed-loop device developed by Ouyang et al. This device integrates EEG, EMG, temperature sensing, optogenetic stimulation, and deep learning-based inference functions into a 2 g subcutaneous module, enabling autonomous seizure suppression and sleep state monitoring by performing optical or pharmacological intervention within 30 ms of detecting seizure activity [94].

In addition, BMI systems that consider multi-party interactions beyond functional closed loops within a single entity are also being proposed. Yang et al. developed wireless multi-party optoelectronic modules that can provide optogenetic stimulation to four rats simultaneously, and which integrate dual-color µ-LEDs, an inertial measurement unit, and short-range communication capabilities into an ultra-lightweight 22 mg head-mounted device, enabling precise synchronization of stimulation patterns at the millisecond level [95]. This allowed them to elucidate the phenomenon of theta wave synchronization between prefrontal cortices, which regulates the social behavior of multiple freely interacting individuals.

Meanwhile, research is continuing to expand spatial resolution within a single organism. Shang et al. implemented a fully flexible cortical display consisting of four 5 × 10 µ-LED arrays and multiplexed fiber bundles, demonstrating that independent photostimulation across a large cortical area is possible with an ultra-lightweight device weighing only 1.3 g [96]. Mechanical stability was ensured through finite element analysis and repeated bending experiments, and precise control was demonstrated in cell-specific stimulation experiments in the prefrontal cortex and lateral habenula without affecting animal behavior. This paves the way for future expansion of holographic-based cortical light stimulation technology to large animals or human models.

Furthermore, Ling et al. proposed a platform for simultaneous stimulation and recording of multiple brain regions using flexible serpentine wiring, liquid crystal polymer substrates, and multifunctional nanocomposites [97]. Through processes such as heterogeneous chiplet assembly, microfluidic-based heat dissipation, and bioadhesive encapsulation, they achieved a tissue-like elasticity of <10 kPa and a structure expandable to over 1000 channels. This strategy is gaining attention as a blueprint for future whole-brain BMI structures.

### 3.4. Technological Development for Improved Biocompatibility

Conventional neural devices face challenges in long-term measurements due to brain tissue damage and inflammatory responses [98,99,100]. Neuroinflammatory responses separate implanted probes from tissues, interfering with long-term brain signal measurements [101].

Consequently, flexible electronic devices with biocompatibility and wireless capabilities have been developed. Jeong et al. developed biomimetic neural probes that dramatically reduce immune responses. Their innovative design employs PEDOT:PSS-coated Au electrodes and SU-8 substrates to implement a super static interface, achieving exceptional biocompatibility for deep brain monitoring and stimulation (Figure 3b) [93].

Soft neural implants such as electronic dura mater (e-dura) probes minimize neuritis and neuron loss by reducing their size and stiffness [100,102,103]. The e-dura developed by Minev et al. presents an innovative design for long-term multimodal neural interfaces, conforming to the spinal cord surface without inducing inflammatory responses [104]. Carbon fiber-based devices are suitable for long-term neural activity recordings with minimal inflammatory responses [105], while mesh electronics (e.g., NeuE) minimize chronic immune responses through mechanical properties similar to biological tissues [106].

Tianhao Chen et al. developed a flexible biodegradable brain stimulation electrode that delivers electrical stimulation for up to seven days after insertion into the body and then naturally decomposes, suggesting the possibility of brain damage recovery and nerve regeneration by inducing the activation of endogenous neural progenitor cells [107]. This device completely degrades within the body without requiring surgical removal after use, making it a next-generation neural regeneration electrotherapy technology that minimizes the risk of inflammation and tissue damage.

Limitations and Challenges: Ultra-soft probes reduce acute trauma, yet chronic micromotion continues to trigger glial scarring that erodes signal quality after 6–12 months in vivo. Real-time impedance tracking has revealed gradual increases of >50% in mesh-type electrodes, suggesting that purely mechanical compliance is insufficient without concurrent anti-inflammatory surface chemistry. In addition, large-area flexible arrays have the problem of complicating the removal of implants and device-tissue separation at the end of use.

### 3.5. Advances in Deep Brain Stimulation

Deep brain stimulation (DBS) has proven to be effective in treating Parkinson’s disease and epilepsy [108,109]. Conventional open-loop systems provide continuous constant stimulation [110], but have limitations in minimizing side effects, as they operate regardless of patient status.

**Figure 4:**
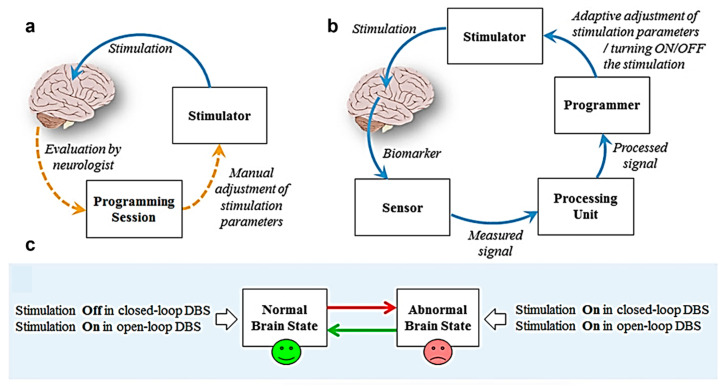
Paradigm shift in deep brain stimulation from open- to closed-loop systems. (**a**) Open-loop DBS provides continuous stimulation with fixed stimulation parameters. (**b**) Closed-loop DBS automatically adjusts based on real-time brain signal monitoring and feedback. (**c**) Comparison of system operation in normal and abnormal brain states; minimizing side effects by providing stimulation only if needed in closed-loop systems based on research by Parastarfeizabadi and Kouzani. Reproduced with permission from [40].

Recently, closed-loop systems that automatically adjust stimulation via real-time monitoring have gained considerable attention [111]. Parastarfeizabadi and Kouzani comprehensively analyzed the development of closed-loop DBS devices and showed that this technology provides superior therapeutic effects and reduced side effects compared with open-loop systems [40]. The differences in the operating principles of these open- and closed-loop systems are presented in Figure 4. Adaptive DBS (aDBS) has shown superior motor symptom improvement than conventional DBS by real-time monitoring of beta-band (13–30 Hz) oscillations [43,112]. Technological advances have improved real-time monitoring reliability, enabling an effective response to long-term changes in patient status [113].

Swinnen et al. point out that while DBS, currently the most widely used treatment for Parkinson’s disease, focuses primarily on improving motor symptoms, non-motor symptoms continue to cause disability in many patients and significantly impact their quality of life [114]. This article emphasizes the insufficient efficacy of existing DBS for non-motor symptoms and proposes the need for adaptive DBS systems to address these symptoms. This demonstrates that neuromodulation strategies capable of real-time responsive control of non-motor symptoms could become a new treatment paradigm in the future.

Limitations and Challenges: Adaptive DBS currently relies on powerful biological markers that are not disease-specific and do not express stably. Beta-band suppression works relatively well for Parkinson’s disease progression, but the electrophysiological correlation with mood, cognitive function, or freezing episodes remains unclear, delaying the expansion of adaptive DBS beyond the motor area. Closed-loop platforms cause system complexity, energy requirements, and shorter charging intervals, which can cause ergonomic burdens on vulnerable patients.

### 3.6. Clinical Translation Pathways and Emerging Applications of Implantable Brain–Computer Interfaces

The clinical transition of implantable brain–computer interfaces follows a highly defined path and requires significant resources and technical expertise. Neuralink’s PRIME study is a prime example, combining robot-assisted implantation surgery with a high-channel wireless telemetry system to shorten the clinical transition timeline. In January 2024, Neuralink successfully implanted the device in its first quadriplegic patient, demonstrating voluntary cursor control and game manipulation capabilities, though challenges remain, including battery management and lead movement issues [115].

The BrainGate study has accumulated over 12,000 days of implant data, successfully implementing functions such as wireless cursor control, speech decoding, and high-bandwidth sensory-motor prosthetic operation in quadriplegic patients [116]. Meanwhile, Synchron has reduced surgical burden and enabled referral-network expansion through a vascular approach that inserts Stentrode via the jugular vein without cranial incision [42]. In the Australian SWITCH study, four patients with severe paralysis completed up to 12 months of follow-up, demonstrating the ability to independently communicate and perform daily digital tasks at home using a fully implanted endovascular brain–computer interface, without any device-related serious adverse events or permanent neurological complication [42].

A recent clinical study by China’s NeuroXess demonstrated that high-density ECoG electrodes, combined with region-specific machine learning algorithms, enabled accurate decoding of spoken language within a few days after surgery, suggesting the potential for rapid functional recovery through brain–computer interface technology. While these cases demonstrate the technical feasibility and commercial potential of brain–computer interfaces, challenges remain in chronic implant environments, including material durability, glial responses to fine movements, the sealing of wireless power transmission, and trade-offs between bidirectional bandwidth and power. Additionally, issues such as lifelong neural data collection conflicting with ethics, privacy, and insurance reimbursement systems require resolution.

### 3.7. Integration of Wireless Technology

The integration of wireless technology with brain-implanted electronic devices is rapidly increasing. Innovative examples, such as Neuralink and Neural Dust, have significantly enhanced the functionality of brain-implanted electronic devices using wireless power and data transmission technology [39,43]. These technologies ensure patient mobility, reduce infection risk, and provide new possibilities for the continuous monitoring and rehabilitation of paralyzed patients.

Fully wireless brain–computer interfaces are raising concerns about privacy, data ownership, and the unexpected neuropsychiatric effects of continuous cortical recording. Regulatory frameworks have yet to define retention periods for high-bandwidth neural data or permissible secondary uses, and early clinical reports describe issues with electrode migration and charging coil mismatches that impair everyday usability. Therefore, standardized human factors testing and transparent risk communication will be essential conditions for mainstream adoption.

## 4. Skin-Implantable Electronic Devices

### 4.1. Various Applications of Skin-Implantable Devices

Skin-implantable electronic devices are used for monitoring biological signals or as drug delivery systems [117,118,119]. Skin-implantable electronic devices are inserted beneath the skin of patients to monitor and treat biological signals. They are useful for managing chronic diseases by continuously measuring important biological signals, such as heart rate, body temperature, and blood glucose levels [120,121].

### 4.2. Drug Delivery Systems

Implantable skin drug delivery systems are increasingly used in pain management, diabetes control, and cancer therapy. These systems address the limitations of conventional oral medications or injectable routes by improving drug stability and eliminating the need for frequent administration, thereby enhancing patient compliance [118]. They significantly increase medication adherence and help maintain consistent therapeutic levels over extended periods. Additionally, such systems improve treatment outcomes and reduce infection risks by enabling precise, programmable control of drug dosage and release timing within the body [119].

### 4.3. Integrated Systems for Diabetes Management

Patients with diabetes often suffer from skin damage, tissue irritation, and microcirculatory complications resulting from repeated blood sampling and frequent insulin injections required for glycemic control [122]. These invasive procedures not only cause physical discomfort but also pose challenges for long-term compliance, especially among pediatric and elderly populations. To address these limitations and reduce the daily burden on patients, hybrid closed-loop systems (HCLS) have emerged as a promising technological solution. These systems integrate continuous glucose monitors (CGMs) with automated insulin delivery (AID) mechanisms to enable real-time, algorithm-driven, autonomous blood glucose regulation [123,124].

**Figure 5:**
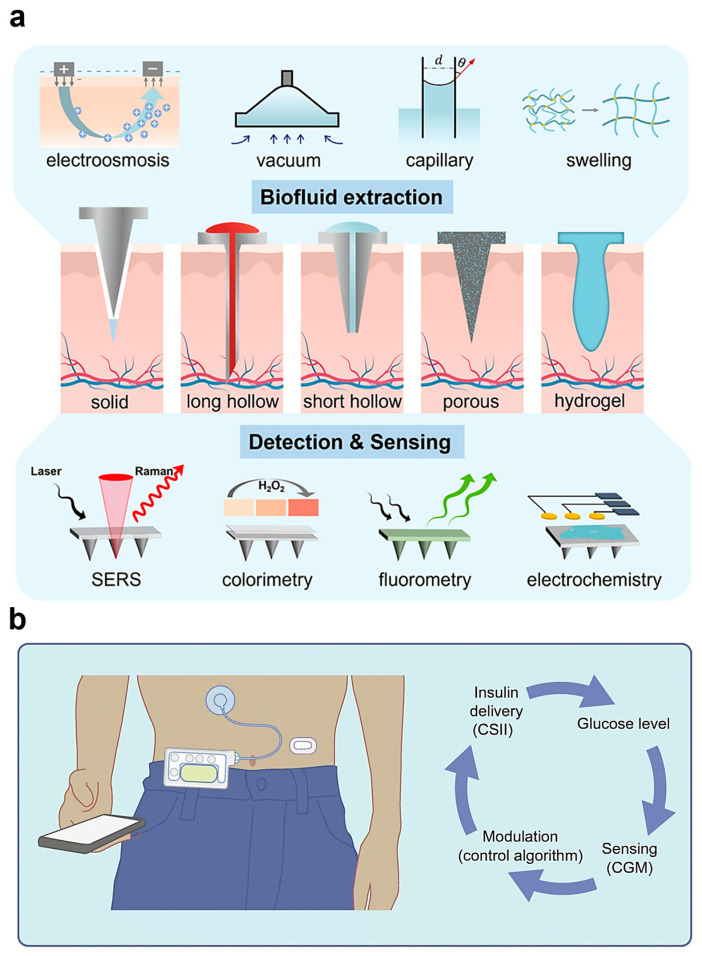
Integrated skin-implantable electronic device systems for diabetes management. (**a**) Conceptual diagram of a microneedle-based glucose monitoring platform. The system enables minimally invasive extraction of biofluids such as sweat or interstitial fluid via microneedles and incorporates multiple sensing modalities including electrochemical detection, fluorescence, colorimetry, and surface-enhanced Raman scattering (SERS) to measure glucose levels. Reproduced with permission from [123]. (**b**) Schematic of a hybrid closed-loop insulin delivery system. Continuous glucose monitoring data are processed by an algorithm that dynamically adjusts insulin infusion in real time, maintaining blood glucose levels within the target range (time in range: 70–80%). Commercialized HCLS devices, such as MiniMed 780G, Tandem Control-IQ, and Omnipod 5 are based on this operational principle. Reproduced with permission from [126].

As illustrated in Figure 5a, recent innovations have introduced microneedle-based glucose monitoring platforms that provide a minimally invasive and user-friendly alternative to conventional finger-prick CGMs [123]. These platforms extract biofluids—such as sweat or interstitial fluid—through micro-scale needles that penetrate the skin without stimulating pain receptors. The collected samples are then analyzed using a variety of sensing techniques, including electrochemical detection, fluorescence emission, colorimetric assays, and surface-enhanced Raman scattering (SERS) [125]. By eliminating the need for frequent blood sampling, these systems are designed to enhance user comfort, reduce infection risk, and support long-term, continuous monitoring, thereby improving adherence and the overall effectiveness of diabetes self-management strategies.

### 4.4. Quantitative Evaluation of Device Performance

Current-generation continuous CGM sensors achieve single-digit mean absolute relative difference (MARD) with minute-scale physiological lag, setting a new clinical benchmark. Dexcom G7 reports a pooled MARD of 8.2% and a lag of 3.5 min, whereas Abbott Libre 3 maintains 8.9% MARD with 1.8 ± 4.8 min lag over a 14-day wear interval [44,45]. For extended wear, implantable and microneedle-based platforms combine low detection limits with multi-month stability. The intradermal Eversense 365 operates for 365 days with MARD of 8.8% [46].

The operating framework of hybrid closed-loop systems is shown in Figure 5b [126]. In these systems, CGMs continuously track glucose fluctuations and feed the data into a control algorithm, which dynamically adjusts the insulin delivery rate in real time. This feedback loop allows for precise and adaptive glycemic control, minimizing both hyperglycemic and hypoglycemic episodes. Several commercialized HCLS products, including MiniMed 780G, Tandem Control-IQ, and Omnipod 5, have demonstrated strong clinical performance by increasing Time in Range (TIR) to approximately 70–80%, which is a key metric for evaluating stable glucose control [127,128]. Beyond improving numerical outcomes, these systems have been shown to significantly enhance the quality of life for individuals with type 1 diabetes, reducing the mental and physical burden associated with daily disease management [129,130]. Furthermore, they consistently outperform traditional multiple daily insulin injection (MDI) regimens in both clinical efficacy and patient satisfaction [131]. Based on this evidence, the American Diabetes Association (ADA) recommended AID systems as a priority for patients with type 1 and other insulin-deficient diabetes in its 2025 guidelines, stating that they can help improve blood glucose control, prevent hypoglycemia, and enhance equity, depending on availability [132].

Limitations and Challenges: Despite impressive Time-in-Range metrics, hybrid closed-loop systems still struggle with sensor drift, algorithm bias, and cost-related inequality issues. Enzyme-based CGM membranes experience approximately a 10% decrease in sensitivity per day of wear, necessitating frequent recalibration, and machine learning dosing algorithms trained on adult datasets may cause excessive corrections for pediatric and pregnant users. Insurance coverage varies widely, and out-of-pocket costs perpetuate inequality, suggesting that technological improvements must be accompanied by policy reforms to realize benefits at the population level.

## 5. Expansion to Gastrointestinal Organs: Biliary Stents and Implantable Electronic Devices

### 5.1. Necessity and Challenges of Developing Gastrointestinal Implantable Electronic Devices

The development of implantable electronic devices for the gastrointestinal system has been challenging because of its deep location and continuous motility. The strongly acidic environment, reaching pH 2 in the stomach or duodenum, poses a significant challenge to material biostability [133,134]. Recently, biodegradable wireless sensors using pH-responsive hydrogels have been developed for the early detection of gastrointestinal leaks [135]. Research on wirelessly powered electrical stimulation devices for the lower esophageal sphincter in patients with reflux esophagitis has been conducted, but remains at the animal testing stage [136]. Considering the high prevalence and mortality rates of gastrointestinal cancers, the importance of this field may increase [137].

### 5.2. Clinical Importance of Bile Duct Cancer and Biliary Obstruction

Among gastrointestinal cancers, bile duct cancer is discovered in advanced stages with a five-year relative survival rate of <3% and distant metastasis [138]. As its incidence is increasing [139], research to improve survival rates is urgently required. Biliary obstruction causes serious complications, such as jaundice, cholangitis, and sepsis [140,141]. Although bypass surgery previously had high complication and mortality rates, endoscopic stent insertion is currently the standard treatment [142].

Stent occlusion can lead to sepsis and death from cholangitis and worsen the prognosis of chemotherapy [143,144,145]. The effective patency period of stents varies from two to six months, making prediction difficult [146,147]. Moreover, no effective method currently exists to confirm stent patency.

### 5.3. Types and Development of Biliary Stents

**Figure 6:**
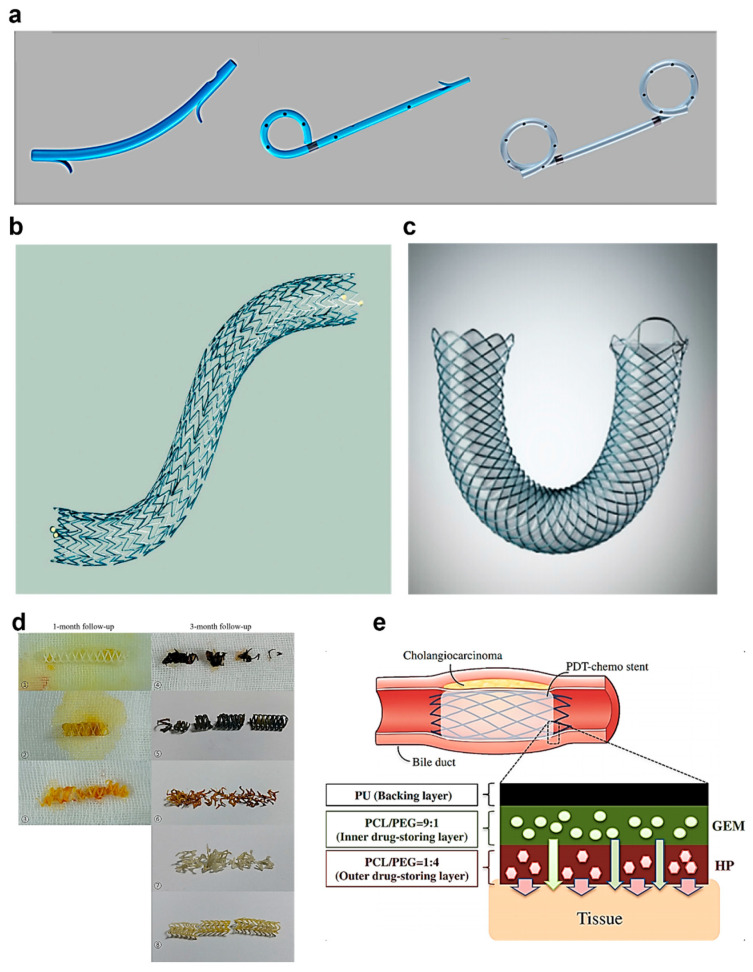
Evolution of biliary stents and development of next-generation smart stents. (**a**) Various types of plastic stents classified by Park and Lee—for short-term biliary drainage. Reproduced with permission from [150]. (**b**) First-generation uncovered self-expandable metal stents reviewed by Moy and Birk—mesh structure facilitating bile drainage. Reproduced with permission from [47]. (**c**) Second-generation covered metal stents—preventing tumor ingrowth with silicone membrane. Reproduced with permission from [153]. (**d**) Three-dimensional-printed biodegradable stents developed by Kim et al.—utilizing biodegradable polymers such as PLGA and PGA. At 1 month, most stents (except specimen 3, which was damaged during extraction) retained their original color and integrity. At 3 months, most stents showed discoloration and fragmentation, (specimens 6, 7, and 8 are prototype stents). Reproduced with permission from [159]. (**e**) Drug-eluting stents developed by Chen et al.—PCL anti-corrosion coating manufactured by electrospinning, integrating photodynamic therapy and chemotherapy drugs. Reproduced with permission from [160].

Biliary stents are inserted through endoscopic retrograde cholangiopancreatography to restore bile flow [148,149]. Park and Lee presented the most appropriate stent selection criteria for endoscopic ultrasound-guided interventions [150]. Plastic stents (Figure 6a) are made of polyethylene, polyurethane, or Teflon for short-term biliary drainage [151] but require replacement every 3–6 months and have limitations for long-term drainage in patients with cancer [152].

According to a comprehensive review of hepatopancreatic stents by Moy and Birk [47], first-generation uncovered self-expandable metal stents (Figure 6b) have a mesh structure made of stainless steel, nickel-titanium, or nitinol [153], but have problems with restenosis and tumor ingrowth [153]. Second-generation covered metal stents (Figure 6c) were attached to silicone membranes but were evaluated as transitional products because of sludge formation and position displacement issues [154,155].

Recent studies have focused on drug-coated biodegradable stents. Drug-coated stents aim to extend stent life with a polycaprolactone (PCL) anti-corrosion coating, silver nano/chitosan antibacterial coating, and paclitaxel/cisplatin anti-tumor coating [156]. Biodegradable stents use PLGA, PGA, and PDX; however, clinical outcomes remain unclear [157,158]. Kim et al. reported that 3D-printed biodegradable biliary stents were safe and feasible in pig models (Figure 6d) [159]. Chen et al. developed a drug-releasing biliary stent prototype combining photodynamic therapy and chemotherapy using electrospinning (Figure 6e) [160].

### 5.4. Development Strategy for Implantable Electronic Devices for Biliary Stents

#### 5.4.1. Current Research Trends

Researchers have attempted ex vivo measurement for biliary stent occlusion prediction by attaching magnetoelastic sensors to plastic stents; however, this approach had limitations for human application owing to distance restrictions within 7.5 cm [161]. In 2024, a study published in Nature Microsystems and Nanoengineering reported that a wireless monitoring system using 28 μm thick magnetoelastic sensors achieved a signal-to-noise ratio of 10^6^ at 17 cm distance in pig models [18].

#### 5.4.2. Pressure Sensor-Based Monitoring

Biliary stents comprise a metal outer layer with a diameter of 8–10 mm and an empty lumen [162], compressed to 2.5 mm in the delivery catheter and expanded in the bile duct [163]. As bile is not drained if the bile duct is obstructed, causing pressure changes [164], stent occlusion can be predicted with pressure sensors. Based on this technical background, there has been recent interest in the development of sensor-integrated stents for real-time pressure detection. For example, there have been reports of wireless pressure sensors embedded in vascular stents to monitor hemodynamic changes in real time [49], suggesting the possibility of applying this technology to bile duct stents. In particular, flexible pressure sensors produced using aerosol jet printing technology provide accurate measurements even at a small bending radius of 0.25 mm, making them suitable for anatomical structures with high curvature, such as bile ducts. Furthermore, Bateman et al. developed an implantable membrane sensor and long-range wireless electronic system capable of real-time monitoring of stent-edge restenosis [50]. This system integrates a low-resistance inductive stent made using laser microfabrication and biocompatible metal plating with a flexible dielectric elastomer-based conformal pressure sensor to precisely detect changes in blood flow pressure at the stent edge. Using a 2 GHz wireless radar method, the system can detect the stent at distances of up to 50 cm and clearly capture frequency changes corresponding to pressure changes even in situations of approximately 50% restenosis. This device demonstrates the potential to open new horizons for wireless stent-based bioelectronic diagnostic platforms in the future.

#### 5.4.3. pH Sensor-Based Monitoring

Bile normally maintains pH 7.5–8.05 but becomes acidic owing to inflammation and bile acid accumulation from biliary obstruction [165,166,167,168]. Silicon nanowire-based pH sensors have been developed and integrated into in-body communication circuits [48], and biodegradable sensors using pH-responsive hydrogels have simultaneously achieved high selectivity and biodegradability [135].

#### 5.4.4. Biofilm Formation Inhibition

Biliary stents become occluded because of bacterial proliferation and biofilm formation from the duodenal fluid reflux [169]. Calcium bilirubinate stones are deposited on the biofilm surface and block the stent lumen [170]. Implantable electronic devices can extend stent life by inhibiting inflammatory responses and bacterial growth using microcurrents or electromagnetic waves.

### 5.5. Preclinical Validation Methods

Pig bile ducts are similar to those in humans, making them suitable for in vivo experiments. Contact with radiofrequency ablation catheters at 80° for 1 min causes biliary stricture after 3–4 weeks [171]. In this model, the sensor functions and wireless data transmission of the developed stents were validated.

## 6. Key Challenges and Future Directions in Implantable Biosensor Technologies

Implantable biosensor technology has recently gained attention as a key means of real-time monitoring of biological signals and implementing precision medicine.However, in order to transition this technology to clinical use, various technical challenges must be overcome, including biocompatibility, miniaturization, power supply, and signal stability.

First, biocompatibility acts as a fundamental obstacle to implantable sensor technology. Implantable sensors inserted into the body for long periods of time inevitably trigger an immune response, and the resulting fibrosis and biofilm formation gradually reduce the sensitivity and reliability of the sensors. Recent approaches to address this issue include the application of soft structures (such as mesh) that align with the mechanical properties of tissues, anti-inflammatory and antimicrobial surface treatments (such as laminin-like PEDOT:PSS), and the development of biodegradable platforms that safely degrade within the body after a certain period [63,172,173]. In the future, it will be necessary to integrate these materials engineering approaches with local release of immunomodulatory drugs and real-time impedance monitoring functionality to enable early intervention before tissue reactions reach critical levels.

In terms of miniaturization, advancements in nanofabrication technology have enabled the production of silicon nanowires as small as 13.5 nm, ultra-small electrodes based on carbon fibers, and flexible electrode patterns using aerosol printing [174,175,176]. However, technical trade-offs still exist in terms of mechanical strength, wireless antenna performance, and on-board computing capabilities. Recently, heterogeneous integration technology that vertically stacks electronic and photonic functions has garnered attention, as it enables a significant improvement in sensing density per unit volume while minimizing cross-sectional area [177,178]. Additionally, the introduction of high-resolution 3D printing and electrospinning technologies tailored to anatomically complex target organs (e.g., bile ducts, cerebral cortex, etc.) could serve as a pivotal turning point in accelerating patient-specific prototyping [179,180].

Power supply issues are a key factor limiting the long-term operation of implantable sensors, and conventional primary batteries are not suitable due to limitations such as size, lifespan, and difficulty in replacement. To address this, a multimodal harvesting approach combining inductive power, ultrasound, photovoltaic, and enzyme-based energy harvesting techniques is being introduced, and efforts are underway to achieve energy autonomy by combining this with ultra-low-power circuits and power-efficient firmware [181,182,183]. In particular, within a local bio-network where multiple implantable devices operate complementarily, power sharing or a method where the main device relays inductive power to auxiliary devices holds the potential for long-term operation without the need for large capacitors. Establishing safety standards for such heterogeneous power transmission technologies will be an essential prerequisite for future clinical approval and commercialization.

Maintaining a high signal-to-noise ratio over long periods of time remains a highly complex challenge for signal stability and data fidelity due to a combination of factors such as electrode corrosion, encapsulation drift, micro-movements of tissue, and electromagnetic interference. Recent strategies to address this include machine learning-based artifact removal algorithms operating locally on ultra-low-power neural processors, and redundancy achieved through spatially distributed sensor arrays capable of withstanding partial node loss [184,185]. In the future, co-design of materials and algorithms is expected to emerge as a core strategy, with hardware–software integration approaches such as real-time compression techniques considering the temporal changes in electrode impedance becoming key technological elements.

Solving technical challenges through continuous technological innovation will enable implantable biosensors to overcome the limitations of traditional laboratory-based prototypes. By integrating innovative technologies that reflect clinical requirements, these sensors are expected to establish themselves as the foundation technology for precision medicine in various fields, including cardiovascular, neuropsychiatric, metabolic, and gastrointestinal diseases.

## 7. Conclusions

Implantable electronic devices are leading innovations in medical technology and have significantly improved patients’ quality of life. Wireless pacemakers, subcutaneous ICDs, and cardiac resynchronization therapy have improved the prognosis of patients with cardiovascular diseases. In the brain, biocompatibility-enhanced flexible electrodes and closed-loop DBS have presented new possibilities for treating neurological disorders. Implantable skin devices have revolutionized diabetes management through hybrid closed-loop systems.

Integrating implantable electronic devices, particularly biliary stents, into the gastrointestinal system is an important direction for future studies. The successful in vivo experimental results of wireless monitoring using magnetoelastic sensors support this hypothesis. Smart stents integrating pressure and pH sensors can improve survival rates for patients with bile duct cancer through early prediction and prevention of biliary obstruction.

Improvements in biocompatibility, long-term stability, and enhanced wireless power and data transmission technologies are necessary for the continued development of implantable electronic devices. The convergence of nanotechnology, materials science, and artificial intelligence could enable the development of sophisticated and intelligent implantable devices.

Implantable electronic devices are changing the modern medicine paradigm. Building on their successful applications in the heart, brain, and skin, these devices are anticipated to expand into other organ systems, including the gastrointestinal system, thereby playing a key role in achieving personalized and precision medicine. Through continuous research and development, we expect to maximize the potential of these technologies to benefit more patients.

## Data Availability

Not applicable.

## Abbreviations

The following abbreviations are used in this manuscript:

ICDs	implantable cardioverter-defibrillators
ICM	implantable cardiac monitors
EEG	electroencephalography
ECoG	Electrocorticography
ECG	Electrocardiogram
LFPs	Local field potentials
SNR	Signal-to-Noise Ratio
DBS	Deep brain stimulation
HCLS	Hybrid closed-loop systems
CGM	Continuous glucose monitors
AID	Automated insulin delivery
PCL	polycaprolactone
PLGA	poly(lactic-co-glycolic acid)
PGA	Polyglycolic acid
PDX	Poly(dioxanone)
PDMS	Polydimethylsiloxane
MARD	Mean Absolute Relative Difference
